# Developing and implementing a protocol for evaluating patient involvement in health technology assessment: the experience of the Spanish HTA network (RedETS)

**DOI:** 10.1017/S0266462326104085

**Published:** 2026-07-29

**Authors:** Eva Reviriego-Rodrigo, Paula Cantero-Muñoz, Laura Llinàs-Mallol, Begoña Rodríguez-Ortiz de Salazar, Vanesa Ramos-García, Analía Abt-Sacks, Matilde Palma-Ruiz, Anabel Granja-Dominguez, Gustavo Mora-Navarro, Beatriz Fernández-Martín, Celia Juárez-Rojo, Ana Toledo-Chávarri

**Affiliations:** 1 Basque Office for HTA (Osteba), Basque Foundation for Health Innovation and Research (BIOEF), Bilbao, Spain; 2 University of the Basque Country (EHU), Faculty of Medicine and Nursing, Department of Preventive Medicine and Public Health, Leioa, Spain; 3 Spanish Network of Agencies for Assessing National Health System Technologies and Performance, Madrid, Spain; 4 Scientific Unit, Galician Health Knowledge Agency (Avalia-t, ACIS), Santiago de Compostela, Spain; 5 Agency for Health Quality and Assessment of Catalonia (AQuAS), Department of Health, Barcelona, Spain; 6 Health Technology Assessment Agency, Carlos III Health Institute, Madrid, Spain; 7 Canary Islands Health Research Institute Foundation, Canary Islands Health Service, Santa Cruz de Tenerife, Spain; 8 Network for Research on Chronicity, Primary Care and Health Promotion (RICAPPS), Tenerife, Spain; 9 Aragon Health Sciences Institute (IACS), Zaragoza, Spain; 10 Andalusian Health Technology Assessment Area (AETSA), Andalusian Public Foundation Progress and Health, Seville, Spain; 11 Health Technology Assessment Unit of the Community of Madrid (UETS), Madrid, Spain; 12 Technical Secretariat of the Spanish Network of Agencies for Assessing National Health System Technologies and Performance, Madrid, Spain; 13 Spanish Scientific Research Council (CSIC), Institute for Advanced Social Studies (IESA-CSIC), Córdoba, Spain

**Keywords:** patient involvement, technology assessment, biomedical, RedETS, Evaluation Protocol, Spain

## Abstract

**Objectives:**

The objective of this study is to describe the development and first implementation of a protocol for evaluating patient involvement in health technology assessment (HTA) within the Spanish HTA Network (RedETS), establishing a framework for continuous monitoring across agencies.

**Methods:**

A multi-phase process informed protocol development. Indicators were proposed and refined based on international evidence and RedETS experience. Three concise questionnaires were designed for agency leads, HTA coordinators, and patients, and improved through consultation and pilot testing. The first evaluation cycle was conducted in March 2025 via online surveys. Quantitative data were summarized descriptively, and qualitative responses analyzed thematically. A stakeholder meeting reviewed findings.

**Results:**

Responses were received from all eight agencies, the coordinators of all forty-nine HTA reports published during the study period, and ten of the thirty-three patients involved in these reports. Twenty reports (41 percent) included patient involvement, mainly through protocol review, draft validation, consultation meetings, or qualitative research. Twenty-nine reports (59 percent) had no patient involvement; among these, six of twenty-nine (21 percent) had attempted recruitment but could not implement it due to recruitment or organizational barriers. In reports with involvement, participation represented a small proportion of assessment time and often relied on a single patient. Patient respondents valued their contribution but noted unclear expectations, technical language, and limited feedback.

**Conclusions:**

The protocol proved feasible and valuable for monitoring whether, how, and when patients are involved in HTA. Strengthening planning, recruitment, and feedback will support more meaningful engagement. RedETS provides a scalable model for continuous monitoring in HTA.

## Introduction

Patient involvement in health technology assessment (HTA) is widely recognized as essential for strengthening transparency, legitimacy, and equity in health policy decision making. The conceptual foundations of patient and public involvement (PPI) – understood as the active contribution of patients, caregivers, and citizens to processes shaping the development and assessment of health technologies – are well established in the literature. Seminal works, including Patient Involvement in HTA (1), EUPATI guidance (2), and HTAi Patient and Citizen Involvement Interest Group (PCIG) position statements (3), emphasize structured and transparent approaches to planning and reporting PPI.

Although several HTA organizations have developed structured procedures for involving patients, international evidence shows that documentation, feedback, and evaluation of patient involvement remain inconsistent and largely project-specific across jurisdictions. Experiences from NICE, HAS, EUnetHTA, and other European initiatives indicate that, while participation is increasingly formalized, mechanisms to monitor how involvement is implemented, sustained, and influences HTA outputs over time remain limited (4–7).

Recent reviews and comparative analyses consistently show that, despite growing interest, the implementation of patient and public involvement in HTA remains heterogeneous, conceptually fragmented, and rarely evaluated systematically or longitudinally. Most initiatives rely on consultation-based approaches, with limited assessment of influence or impact, and evaluation activities are often confined to isolated projects or single assessments (8–13). Against this backdrop, the RedETS initiative represents a rare example of a national HTA network implementing structured, coordinated, and recurrent evaluation of patient involvement.

The Spanish Network of Agencies for Assessing National Health System Technologies and Performance (RedETS) has progressively strengthened patient involvement by developing methodological guidance, decision-support tools, and practical resources to support its implementation across agencies. Foundational RedETS documents – such as the methodological manual for patient involvement (14), the decisional flowchart that guides when and how patients should be involved in HTA (15;16), and the national assessment of patient involvement in HTA reports published in 2021 (17) – have contributed to standardizing expectations and promoting more coherent practices within the network. Insights from Spanish HTA researchers further highlight areas of progress, persistent challenges, and opportunities to enhance the meaningful and systematic incorporation of patient perspectives in HTA processes (18).

To consolidate these advances and strengthen accountability, RedETS launched an initiative to institutionalize annual monitoring of patient involvement across all member agencies. This article describes the development and first implementation of the RedETS monitoring protocol, illustrating a replicable model to foster continuous improvement and shared learning across the network. The first annual evaluation revealed variability in implementation and operational challenges that reinforce the need for structured longitudinal monitoring.

## Methods

The evaluation protocol was developed through a three-stage process involving the Evaluation Service of the Canary Islands Health Service (ESCIHS) and the RedETS Interest Group on Patient Involvement and Social Determinants of Health (IG-PISDH), which includes representation from all eight HTA agencies and units within the RedETS network.

### Development of evaluation indicators

The evaluation methodology was informed by a literature review identifying existing frameworks for assessing patient and public involvement in HTA and related contexts (17).

Identified frameworks were assessed by the research team according to predefined criteria, including conceptual relevance, adaptability to the HTA context, feasibility of implementation, and capacity to support systematic and longitudinal evaluation. Based on these criteria, the Patient Engagement Monitoring and Evaluation Framework developed within the *Patients Active in Research and Dialogues for an Improved Generation of Medicines* (PARADIGM) project was selected as the reference framework (19).

The PARADIGM framework was adapted to the RedETS context through a two-round modified Delphi study aimed at selecting and prioritizing indicators for evaluating patient involvement in HTA reports. Participants were multidisciplinary stakeholders with experience in HTA and patient involvement, representing all agencies within the RedETS network.

In each Delphi round, participants rated the relevance of each indicator using a ten-point Likert scale. Indicators were prioritized based on median scores, and consensus was assessed using interquartile ranges (IQR), with an IQR ≤ 2 considered indicative of adequate agreement. Following the Delphi process, a methodological workshop involving the core research team and external collaborators was conducted to translate the prioritized indicators into an operational evaluation strategy. This workshop focused on defining evaluation objectives, aligning indicators with data collection methods and structuring the evaluation timeline.

Full methodological details of the Delphi process are reported elsewhere (17).

### Questionnaire design and pilot testing

Three complementary questionnaires were developed, each tailored to a specific respondent group and designed to capture the dimensions most relevant to their role in the HTA process. The questionnaire for agency leadership explored organizational structures, governance mechanisms, available resources, and structural enablers or barriers to patient involvement. The questionnaire for HTA report coordinators focused on implementation strategies, practical challenges encountered during the assessment process, and the extent to which patient contributions were integrated into reports. Finally, the patient questionnaire examined participants’ motivations, lived experiences, understanding of the process and perceived influence on the final HTA outputs.

The initial indicators that shaped the questionnaires resulted from an in-depth analysis and systematic work, documented in a foundational methodological report coordinated by ESCIHS (17). Building on this groundwork, the first drafts of the questionnaires were developed and refined through a two-round internal consultation process involving representatives from RedETS agencies and patient organizations.

These drafts, informed by ESCIHS indicators and prior RedETS experience, were further refined through a collaborative review process with the participation of agency leads, report coordinators, and patients.

### Protocol development

An initial version of the protocol guided the first evaluation cycle, and the consolidated version of the “*Protocol for the Implementation of the Evaluation of Patient Involvement in HTA Reports*” was finalized in May 2025.

The protocol defines the scope, data collection methods, responsibilities, and analysis plans. The protocol was validated by the RedETS Secretariat and IG-PISDH members, and includes guidance on longitudinal data collection, timing of distribution, and integration with the RedETS information systems.

To enhance transparency, reproducibility and international comparability, the final validated versions of the three questionnaires currently in use (for agency leads, HTA report coordinators and participating patients) are provided as Supplementary Materials (Supplementary Files S1–S3). These instruments incorporate refinements introduced after the initial pilot cycle and reflect the consolidated evaluation framework adopted by RedETS. The full implementation protocol is provided as Supplementary File S4.

### Implementation in the first annual cycle

All RedETS agencies were invited to participate in the first evaluation cycle, following harmonized guidance on respondent selection and timelines. A central coordination team monitored response rates, ensured consistency across agencies, and provided technical support throughout the process.

The three questionnaires were administered electronically through the survey platform “*Encuesta Fácil*” (Madrid, Spain), each directed to a specific respondent group. Agency leads completed one questionnaire per agency or HTA unit, providing information on organizational structures and support for patient involvement.

HTA report coordinators completed one survey for each of the forty-nine HTA reports published during the study period, from January 2024 to February 2025, consolidating the relevant information for their respective assessments. Patients who had been involved in these reports were invited to complete a dedicated patient questionnaire, designed to capture their experiences and perceived contributions.

Data collection for the first annual cycle took place in March 2025, ensuring a consistent reference period across agencies and respondent groups.

### Data analysis

Quantitative data were analyzed using descriptive statistics. Qualitative data derived from open-ended questionnaire responses were analyzed using a thematic, descriptive–interpretative approach, consistent with the evaluative and applied purpose of the study. Initial coding of qualitative responses was conducted by the central coordination team responsible for the evaluation. To enhance analytical rigor, emerging codes, categories, and preliminary themes were subsequently reviewed and discussed within a multidisciplinary analysis group that included HTA experts and representatives of the RedETS IG-PISDH.

Discrepancies in coding or interpretation were addressed through iterative, consensus-based discussions within the analysis group, leading to refinement and validation of the final thematic structure. Analytical triangulation was applied by comparing findings across respondent groups (HTA agency leads, report coordinators, and participating patients) and across agencies, allowing the identification of convergent and divergent patterns. This triangulation process strengthened the credibility and trustworthiness of the qualitative findings and supported cross-validation of results.

### Stakeholder discussion and adaptation

A stakeholder meeting in May 2025 brought together the RedETS Secretariat and selected representatives from the IG-PISDH. Based on the findings, adjustments were introduced to improve recruitment guidance, clarify items, and strengthen feedback mechanisms.

### Ethical considerations

According to local regulations, this project was considered a service evaluation/quality improvement initiative and did not require formal ethics committee approval. The work was conducted within the framework of the annual activities of RedETS and funded as part of its annual activities commissioned by the Spanish Ministry of Health. Participation was voluntary, and data were analyzed in an anonymized form.

## Results

### Development of evaluation indicators

A comprehensive literature review identified 15 relevant studies out of 85 screened references. Based on this review, the PARADIGM framework (19) was selected and adapted to the RedETS context through a two-round Delphi process (forty-seven and thirty-eight participants, respectively), resulting in thirty-five indicators across structural, process, and outcome dimensions, which formed the empirical basis for the three annual questionnaires administered to HTA agency leads, report coordinators, and participating patients. These indicators supported the implementation of a dual evaluation strategy combining annual monitoring with a quinquennial case study.

### Pilot testing

In 2024, a pilot test was conducted with five agency leads, five HTA report coordinators, and four patients to assess the clarity, coherence, and usability of the questionnaires. While patients reported that the instruments were clear and relevant, agency leads and coordinators provided methodological feedback that led to substantial refinements, including simplification of language, redesign of filter questions, removal of redundant items, improved introductory texts, and clarification of data protection and consent procedures. The revised questionnaires were subsequently uploaded to the online survey platform for final usability testing. The outcomes of the pilot test also informed the development of the first draft of the evaluation protocol.

### Implementation in the first annual cycle

The first annual evaluation cycle was implemented in March 2025 using the three online questionnaires directed at agency leads, HTA report coordinators, and participating patients. Surveys were administered through the platform “*Encuesta Fácil*” (Madrid, Spain). Responses were obtained from all eight RedETS agencies and from the coordinators of all forty-nine HTA reports published during the study period, from January 2024 to February 2025. In addition, ten of the thirty-three patients involved in these reports completed the patient questionnaire.

The first cycle also revealed several methodological aspects requiring refinement. In particular, it became clear that reports with no planned involvement needed to be distinguished from reports in which involvement had been planned but could not be implemented. Additional improvements were required to enhance the clarity and consistent application of the RedETS decisional algorithm (15;16), as well as to ensure that patient questionnaires were systematically distributed immediately after publication of the corresponding HTA report. These insights informed targeted adjustments to strengthen future evaluation cycles and improve the robustness and comparability of longitudinal monitoring.

During the first annual evaluation cycle, all HTA reports published by RedETS within the defined study period were included in the analysis. A total of forty-nine HTA reports were assessed, of which twenty reports (41 percent) incorporated patient involvement. Across these reports, thirty-three patients were involved in participatory activities. Of these, ten patients completed the patient questionnaire (30 percent). The lower response rate among patients was primarily attributable to the timing of questionnaire distribution, which in several cases occurred months after participation. This methodological limitation was identified during the first cycle and has been addressed in the revised protocol, which stipulates that patient questionnaires are to be sent immediately after publication of the HTA report.

#### 1. Agency leadership (8/8; 100 percent)

All eight RedETS agencies completed the questionnaire, providing a comprehensive overview of organizational readiness for patient involvement. Overall, responses reflected a growing institutional commitment to integrating patient perspectives into HTA processes. However, the degree of implementation varied notably across agencies. Several agencies reported having designated staff or structural mechanisms to support patient involvement, yet dedicated financial resources and formal training programs remained unevenly available.

Most agency leads recognized the strategic value of patient involvement in enhancing transparency, accountability, and the relevance of HTA outputs. Nonetheless, they also identified persistent challenges that limit consistent implementation. These included insufficient staff time, competing organizational priorities, limited specialized expertise in patient engagement, and weak mechanisms for systematic interagency collaboration. Despite these barriers, agencies expressed strong support for maintaining the annual evaluation and emphasized the need for harmonized, long-term strategies across the RedETS network to consolidate and strengthen patient involvement practices.

#### 2. HTA report coordinators (49/49; 100 percent)

The evaluation cycle gathered responses for forty-nine HTA reports published between January 2024 and February 2025. Surveys were completed by the respective report coordinators or main technical staff, reflecting strong engagement and a shared interest in advancing patient involvement within RedETS. To characterize current practices and barriers, results were analyzed in two categories: reports without patient involvement and reports with patient involvement.

##### 2A. Reports without patient involvement (29/49; 59 percent)

Twenty-nine of the forty-nine reports assessed did not incorporate patient involvement. Two distinct situations were identified among these twenty-nine reports:

**Participation not planned (23/29; 79 percent; 47 percent of all reports).** Patient involvement had not been considered or integrated into the assessment protocol.

**Participation planned but not implemented (6/29; 21 percent; 12 percent of all reports).** Coordinators had applied the RedETS decisional algorithm and contacted patient organizations, but participation could not be implemented due to recruitment difficulties, lack of responses from patients or organizations, insufficient time within the assessment workflow, or limited institutional support.

Several coordinators reported that early efforts (e.g., outreach activities and initial planning) were insufficient to overcome operational constraints. Although some attributed the absence of involvement to the nature of the technology, the RedETS decisional algorithm states that patient involvement should always be considered and only exceptionally ruled out with explicit justification.

These planned-but-unsuccessful attempts illustrate a growing awareness of the importance of patient perspectives, while highlighting persistent structural and operational barriers that must be addressed to make patient involvement feasible and sustainable across agencies.

##### 2B. Reports with patient involvement (20/49; 41 percent)

Twenty of the forty-nine HTA reports assessed incorporated some form of direct patient involvement. Involvement was generally concentrated at specific stages of the HTA process rather than embedded throughout the whole assessment. The most frequent activities were protocol review and validation of the preliminary draft report. Other forms of direct involvement included consultation meetings and, in a smaller number of cases, primary qualitative research, such as interviews or other approaches aimed at collecting patients’ experiences and perspectives. In parallel, some HTA reports incorporated patient perspectives indirectly through the review or synthesis of qualitative evidence on patient experiences and preferences; however, this was considered distinct from direct patient involvement.

In seventeen of these twenty reports (85 percent), patient involvement accounted for <25 percent of the total assessment time. Most reports relied on a single patient, usually identified through patient organizations, and only one report involved inter-agency collaboration to support patient engagement. Very few assessments included primary qualitative research or systematic reviews of qualitative evidence on patient experiences and perspectives.

Key dimensions of meaningful involvement, such as participant diversity, recognition of contributions, training, and the accessibility of materials, were inconsistently addressed. Some coordinators reported concrete impacts, including adjustments to report content or the inclusion of underrepresented patient needs. However, in other cases, contributions were perceived as having limited influence on key decisions.

Overall, the findings suggest that patient involvement was feasible and valued, but still limited in intensity, continuity, and methodological depth. Recurrent challenges included unclear expectations, technical language, limited feedback mechanisms, recruitment difficulties, insufficient institutional support, and tight production timelines. Respondents emphasized the need for clearer participation protocols, practical recruitment tools, capacity-building opportunities, and more structured two-way communication throughout the HTA process.

#### 3. Participating patients (10/33; 30 percent)

Thirty-three patients had been involved in the forty-nine HTA reports assessed. Of these, ten completed the patient questionnaire. Overall, respondents reported positive experiences and considered their contributions meaningful. Their main motivations for participating included the desire to improve healthcare quality, share their lived experiences and support others facing similar conditions.

Despite these positive perceptions, patients identified several areas for improvement. They reported difficulties understanding technical terminology used in HTA reports, uncertainty about their specific role within the assessment process, and insufficient follow-up or feedback on how their input influenced the final outputs. Patients recommended clearer communication from the outset, earlier involvement in the assessment process, more accessible materials, explicit recognition of their contributions, and structured two-way communication before, during, and after participation.

Sociodemographic information on participating patients was collected in a limited and heterogeneous manner and did not allow a robust assessment of participants’ representativeness in terms of gender, socioeconomic position, or other social determinants of health.

This 30 percent response rate suggests challenges in sustaining engagement over time and underscores the need to distribute the patient questionnaire immediately following publication of the relevant HTA report to maximize recall, relevance, and response rates.

The insights provided by patients directly informed several refinements to both the protocol and the questionnaires, supporting greater methodological consistency and promoting more patient-centered implementation in future evaluation cycles.

## Discussion

This study presents the first structured, system-level approach to monitoring patient involvement in HTA within the RedETS. The development and implementation of a shared, evidence-informed protocol – codesigned with key stakeholders and applied consistently across agencies – represents an important step toward consolidating patient involvement as a routine, transparent, and accountable component of HTA practice. By integrating validated indicators, tailored questionnaires, and a coordinated annual data collection strategy, the RedETS initiative provides a transferable and replicable framework for embedding systematic evaluation within routine HTA processes, contributing to the methodological consolidation of patient involvement evaluation at network level.

Findings from the first annual evaluation cycle show progress in the institutionalization of patient involvement, alongside marked variability in its implementation across agencies and reports. In many assessments, patient engagement occurred late in the process and was limited in scope or influence. Structural and organizational constraints – such as tight timelines, limited staff capacity, and inconsistent use of the decision-support algorithm – continue to restrict opportunities for earlier and more meaningful involvement, highlighting the need for improved planning and stronger institutional support across the HTA life cycle.

The observed heterogeneity across agencies is consistent with implementation science literature showing that early adoption of organizational innovations typically follows uneven trajectories before stabilization. From this perspective, variability may reflect differential levels of organizational readiness and resource allocation rather than conceptual disagreement regarding the value of patient involvement. Longitudinal monitoring will be essential to assess whether greater convergence emerges over successive evaluation cycles.

Triangulation of perspectives from agency heads, HTA report coordinators, and patients provided a multilayered understanding of current practices. While patients valued the opportunity to contribute, they reported difficulties related to technical language, unclear expectations and limited feedback on how their input influenced final reports. Coordinators identified similar challenges, particularly in recruitment and the absence of practical guidance for planning engagement. These convergent findings point to the need for more accessible materials, clearer communication strategies, and formalized feedback mechanisms to enhance both the quality and perceived value of patient involvement. They also support the further development and expansion of structured HTA training initiatives aimed at patients and the general public, already implemented across several RedETS agencies, as a means of strengthening informed and meaningful participation.

In addition, patient involvement may be perceived as introducing additional procedural steps in contexts characterized by strict timelines and efficiency pressures. Balancing meaningful deliberation with procedural efficiency remains a structural challenge for HTA agencies, particularly in resource-constrained settings. System-level monitoring can help make these tensions visible and support more realistic planning of engagement activities, integrating participation as a planned component rather than an add-on to HTA processes.

The RedETS experience aligns with international evidence showing that, despite widespread policy commitments to PPI, systematic and recurrent evaluation remains uncommon across HTA systems. Most published evaluations are scarce, heterogeneous, and often limited to single-case analyses, with evaluation activities frequently conducted on an ad hoc or assessment-specific basis (20). In this context, the RedETS monitoring protocol contributes a distinctive network-level perspective by embedding evaluation as a routine activity across a national HTA network, enabling comparative analysis, identification of structural barriers, and longitudinal monitoring of progress over time.

Recent European regulatory developments further underline the relevance of such approaches. Regulation (EU) 2021/2282 establishes Joint Clinical Assessments (JCAs) as a core component of the future European HTA framework and includes a legal mandate for patient involvement. However, it leaves open key operational questions regarding how participation should be implemented, documented, and evaluated across Member States (21;22). In this context, system-level monitoring tools such as the RedETS protocol may provide a practical evidence base to support patient involvement requirements within future JCAs.

Importantly, the RedETS protocol was designed to be adaptable rather than prescriptive. Its core components – use of a recognized evaluation framework, consensus-based indicators, tailored instruments for different stakeholder groups, and recurrent data collection – can be adopted incrementally and adapted to different institutional contexts and resource levels. This flexibility supports transferability to other HTA systems and aligns with international calls to foster a culture of evaluation and continuous improvement in patient involvement, while avoiding tokenistic practices (23). The first cycle also showed that a shared monitoring framework can strengthen consistency across agencies while preserving the possibility of refining indicators, recruitment procedures, and patient-adapted HTA training initiatives over successive cycles.

The recent publication of the second international edition of Patient Involvement in HTA (24), including a dedicated chapter on the Spanish RedETS experience (25), reflects the growing maturity of the field while also underscoring the persistent need for standardized approaches to evaluating patient involvement. In this context, the present study contributes empirical evidence on how patient involvement can be monitored as a continuous, system-level process within a national HTA network, moving evaluation beyond isolated assessments toward a recurrent mechanism for transparency, accountability, and continuous improvement.

Several limitations should be acknowledged. The evaluation relies partly on self-reported data; however, triangulation across respondent groups mitigates this risk. Although thirty-three patients participated across twenty HTA reports, only ten completed the patient questionnaire, reflecting operational limitations of the first evaluation cycle. This directly informed a procedural refinement in the revised protocol, which now mandates questionnaire distribution immediately after report publication.

Challenges related to diversity and representativeness also emerged. Participation was based predominantly on representatives of patient organizations, with limited involvement of individual patients or caregivers. We acknowledge that this pattern tends to favor participants with greater organizational capacity or health literacy, potentially leading to the under-representation of more socially or structurally vulnerable groups whose capacity to engage in formal HTA processes may be constrained by contextual factors. Although declarations of interest were required and no conflicts were reported, the limited number of participants and the predominance of organizational representatives warrant cautious interpretation.

Recent debates emphasize that patient involvement is not neutral and may privilege certain profiles over others. Sociological analyses highlight how “expert patients” are often more readily legitimized in deliberative forums (26), while concerns have been raised about conflicts of interest and equity implications in HTA participation (27). From a critical perspective, participation has also been conceptualized as a political activity shaped by power relations and institutional contexts. Furthermore, the way patients participate has evolved from a passive model to one in which patients identify problems and set priorities, thereby transforming the relationship between the state and society. In RedETS, patient involvement was mainly advisory and concentrated on specific stages of the assessment process, underscoring the importance of monitoring not only the presence of participation but also its scope and potential influence.

Several authors have highlighted that patient involvement in HTA is not a neutral activity and may raise issues related to representation, legitimacy, and the distribution of influence within assessment processes. Current HTA literature emphasizes that who participates, at what stage, and with what degree of influence can shape both deliberation and outcomes (24). In this context, the RedETS protocol is not intended to resolve these normative questions, but to provide a structured mechanism to systematically document how participation is implemented in practice. By supporting transparency, reflexivity, and longitudinal analysis, it offers a robust foundation for future improvements. Priority areas include earlier integration of patient involvement, strengthened recruitment pathways, enhanced methodological support for coordinators, and improved communication and feedback structures. In addition, expanding structured HTA training initiatives aimed at patients and citizens may help strengthen individual-level participation, improve understanding of coverage decision-making processes, and reduce inequities linked to the underrepresentation of vulnerable groups.

Overall, the RedETS experience contributes novel empirical evidence and offers a scalable model for other HTA systems seeking to institutionalize structured, transparent, and meaningful patient involvement. Ultimately, institutionalizing evaluation may represent one of the most effective strategies for transforming patient involvement from a normative principle into a measurable and accountable component of HTA systems.

## Conclusion

The 2025 evaluation cycle marks a decisive step in consolidating patient involvement as a core component of HTA practice within RedETS. The implementation of a shared, codesigned, and evidence-informed protocol has established a structured foundation for sustained annual monitoring, providing a baseline that highlights both progress and persistent structural and methodological challenges.

The findings underscore the need to strengthen early planning, recruitment strategies, methodological support, and feedback mechanisms to ensure more meaningful and influential engagement across the HTA life cycle. They also show the value of monitoring not only whether patients are involved, but also how and when they are involved and to what extent their contributions may influence the HTA process.

The monitoring system introduced through this protocol represents an innovative contribution to the international HTA landscape, where longitudinal and system-level approaches to evaluating patient involvement remain limited. In this context, the RedETS experience offers a scalable and adaptable model for institutionalizing structured, transparent, and accountable participatory evaluation.

## Supporting information

10.1017/S0266462326104085.sm001 Reviriego-Rodrigo et al. supplementary material Reviriego-Rodrigo et al. supplementary material

## Supplementary material

The supplementary material for this article can be found at http://doi.org/10.1017/S0266462326104085.
