# Supplementary material for: Developing and implementing a protocol for evaluating patient involvement in health technology assessment: the experience of the Spanish HTA network (RedETS)
Source: Int J Technol Assess Health Care. 2026 Jul 29;42(1):e76. doi: 10.1017/S0266462326104085 (PMC13735836; doi:10.1017/S0266462326104085)
Supplement: Supplementary file 1 [file S0266462326104085sup001.zip › Supplementary_File_S2_Questionnaire_Coordinators_HTA_Reports_Revised_English.docx]

**Supplementary File S2. Questionnaire for Coordinators of HTA Reports**

Spanish Network of Agencies for Assessing National Health System Technologies and Performance (RedETS)
Annual Evaluation of Patient Involvement in HTA Reports
2025 Version

**Cover Letter**

**Subject:** Annual Questionnaire on Patient Involvement in HTA Reports (RedETS)

Dear HTA Report Coordinators,

We invite you to participate in the annual questionnaire addressed to coordinators of Health Technology Assessment (HTA) reports developed within the Spanish Network of Agencies for Assessing National Health System Technologies and Performance (RedETS).

One questionnaire must be completed for each report published between [date] and [date], regardless of whether patient involvement took place during its development.

In cases where patient involvement was attempted but could not be achieved, it is important to report this information, as it is essential for identifying barriers and opportunities for improvement.

The information collected will provide an overall view of the level and characteristics of patient involvement in HTA reports and its perceived impact on the assessment process.

Including the voice and lived experience of patients contributes to enhancing the quality, legitimacy, and usefulness of HTA reports.

Estimated completion time: approximately 10 minutes.
Deadline for submission: [day] [month].
Access the questionnaire here: [[link](https://www.encuestafacil.com/respweb/cuestionarios.aspx?EID=2962425)]

Responses will be treated confidentially and used exclusively for evaluation and continuous quality improvement purposes.

We sincerely appreciate your collaboration.

Sincerely,
[Signature]
[Name of the Agency/Unit]

**Questionnaire**

Please complete one questionnaire for each HTA report coordinated and published during the evaluation period, regardless of whether patient involvement was implemented.

**Section 1. Report Identification**

**Q1. Full name:**
**Q2. Agency/Unit:**
**Q3. Title of the HTA report:**
**Q4. Year of publication:**

**Section 2. Planning and Organisational Context**

**Q5. Was patient involvement planned at the protocol stage?**
☐ Yes
☐ No

**Q6. Estimated proportion of total report development time devoted to patient involvement:**
☐ <25%
☐ 25–50%
☐ 50–75%
☐ >75%
☐ Not applicable

**Q7. Did your agency/unit collaborate with other RedETS agencies on patient involvement for this report?**
☐ Yes
☐ No

**Q7a. If yes, please specify the agencies involved and the nature of the collaboration:**

**Q8. Did your agency/unit collaborate with other RedETS agencies on qualitative research related to patient involvement?**
☐ Yes
☐ No

**Q8a. If yes, please specify the agencies involved and the nature of the collaboration:**

**Q9. Was the patient involvement decision-making algorithm applied in this report?**
☐ Yes
☐ No
☐ Not applicable

**Section 3. Implementation of Patient Involvement**

**Q10. Were patients involved in the development of this report?**
☐ Yes
☐ No

**Q10a. If no, please indicate the main reasons for the absence of patient involvement:**

*Note: If patients did not participate in the development of this HTA report, the questionnaire ends after this question. The remaining questions apply only to reports that included patient involvement.*

**Q11. How were patients identified for participation?** (Select all that apply)

☐ Patient organisations
☐ Healthcare professionals
☐ Online platforms or social media
☐ Snowball sampling
☐ Direct selection by the research team
☐ Other (please specify): _____________

**Q12. At which stages of the HTA report were patients involved?** (Select all that apply)

☐ Working group meetings
☐ Scoping / definition of research questions
☐ Protocol review
☐ Evidence review
☐ Primary research study
☐ Analysis or interpretation of results
☐ Draft report review
☐ Other (please specify): _____________

**Q13. Total number of patients involved in the report:**

**Q14. Was a systematic review of qualitative or mixed-methods studies conducted?**
☐ Yes
☐ No

**Q14a. If yes, were patient-experience studies identified and used in the report?**

**Q15. Was primary research conducted to collect patients’ experiences, values, or preferences?**
☐ Yes
☐ No

**Q15a. Methods used (select all that apply):**
☐ Focus groups
☐ Interviews
☐ Surveys
☐ Other (please specify): _____________

**Q16. Types of participants involved:** (Select all that apply)

☐ Individual patients
☐ Expert patients
☐ Patient organisation representatives
☐ Caregivers or family members
☐ Citizens or members of the general public
☐ Other (please specify): _____________

**Q17. Were conflicts of interest declared by participating patients?**
☐ Yes
☐ No
☐ Not applicable

**Q17a. Types of conflicts declared (select all that apply):**

☐ Industry-funded travel or meeting support
☐ Speaking fees
☐ Educational funding
☐ Research funding
☐ Consultancy
☐ Equity or financial interests
☐ Other (please specify): _____________

**Section 4. Diversity, Recognition, and Feedback**

**Q18. Were diversity criteria considered when selecting participants?**
☐ Yes
☐ No

**Q18a. If yes, please specify the criteria applied and their contribution:**
**Q18b. Please describe any barriers or facilitators related to diversity representation:**

**Q19. How was patient contribution acknowledged in the report?** (Select all that apply)

☐ Authorship
☐ Advisory role
☐ Internal review
☐ External review
☐ Acknowledgements

**Q20. Number of participating patients offered HTA training:**
**Q20a. Number who completed the training:**

**Q21. Were plain-language materials developed to support patient participation?**
☐ Yes
☐ No

**Q21a. If yes, please specify the type of materials developed:**

**Q22. Were patients asked whether they understood the information provided?**

☐ Never
☐ Rarely
☐ Sometimes
☐ Frequently
☐ Always

**Q23. What type of feedback did participating patients receive for their collaboration?** (Select all that apply)

☐ Accessible summary report
☐ Formal acknowledgement in the report
☐ Individual meeting or communication
☐ None
☐ Other (please specify): _____________

**Q24. What type of recognition was given to patients in the report?** (Select all that apply)

☐ Authorship
☐ External review
☐ Internal review
☐ Advisory role
☐ Acknowledgements
☐ Other (please specify): _____________

**Section 5. Impact and Lessons Learned**

**Q25. What impact did patient involvement have on this report?**

**Q26. Did patient involvement lead to changes in the report?** (Select all that apply)

☐ Changes in the formulation of the research question (PICO)
☐ Changes related to intervention acceptability
☐ Implementation considerations
☐ Identification of future research needs
☐ No changes
☐ Other (please specify): _____________

**Q27. What barriers did you identify to effective patient involvement?**

**Q28. What facilitators enabled patient involvement in this report?**

**Q29. What suggestions would you propose to improve patient involvement in future HTA reports?**

**End of Questionnaire**

Thank you for your time and valuable collaboration. Your responses will contribute to improving the integration of patient perspectives in Health Technology Assessment.

Technical Secretariat
Spanish Network of Agencies for Assessing National Health System Technologies and Performance (RedETS)
