# Supplementary material for: Developing and implementing a protocol for evaluating patient involvement in health technology assessment: the experience of the Spanish HTA network (RedETS)
Source: Int J Technol Assess Health Care. 2026 Jul 29;42(1):e76. doi: 10.1017/S0266462326104085 (PMC13735836; doi:10.1017/S0266462326104085)
Supplement: Supplementary file 1 [file S0266462326104085sup001.zip › Supplementary_File_S3_Questionnaire_Patients_Revised_English.docx]

**Supplementary File S3. Questionnaire for Patients Participating in HTA Reports**

Spanish Network of Agencies for Assessing National Health System Technologies and Performance (RedETS)
Annual Evaluation of Patient Involvement in HTA Reports
2025 Version

**1. Internal Email – Instructions for HTA Report Coordinators**

**Subject:** Questionnaire for Patients Participating in HTA Reports – RedETS

Dear colleagues,

Please find attached the cover letter and the link to the questionnaire addressed to patients who have participated in Health Technology Assessment (HTA) reports developed within RedETS.

**What is required?**

Each time an HTA report involving patient participation is published, please send the questionnaire to the participating patient(s), as you have been their main contact person throughout the process.

The purpose is to collect feedback on their experience of participating in that specific report, as close as possible to the completion of the assessment process.

**Before sending the questionnaire:**

- Include the patient’s name at the top of the cover letter.
- Sign the letter with your name, position, and contact details.
- Send the letter together with the link to the questionnaire:
  [[Patient Questionnaire link](https://www.encuestafacil.com/respweb/cuestionarios.aspx?EID=2962426)]

Responses will be collected on a rolling basis as reports involving patient participation are published.

Thank you for your collaboration and for helping to maintain this evaluation process.

Kind regards,
[Name and surname]
[RedETS Agency/Unit]

**2. Cover Letter to Be Sent to the Patient**

**Subject:** Questionnaire about your participation in the Health Technology Assessment report – RedETS

Dear [Patient’s Name],

We would like to invite you to complete a short questionnaire about your experience participating in the Health Technology Assessment (HTA) report in which you recently contributed. The report was developed by the Spanish Network of Agencies for Health Technology Assessment and Services of the National Health System (RedETS).

The purpose of this questionnaire is to understand your views on how the perspectives of patients, family members, or citizens were incorporated into this specific report.

Your feedback will help us improve the way patients’ experiences and needs are integrated into future HTA processes.

**Practical information**

Estimated completion time: approximately 10 minutes
Confidentiality: Your responses will be treated confidentially and used exclusively for evaluation and continuous quality improvement purposes.
Deadline for response: [insert date or “within the next 15 days”]
Access the questionnaire here: [[Patient Questionnaire link](https://www.encuestafacil.com/respweb/cuestionarios.aspx?EID=2962426)]

If you have any questions or require further information, please do not hesitate to contact me.

Thank you very much for your time and valuable contribution.

Sincerely,
[Name and surname of the coordinator]
[Name of the Agency/Unit]

**Questionnaire**

**Page 1 – Welcome**

Thank you for participating in this evaluation of patient involvement in Health Technology Assessment (HTA) reports developed by the Spanish Network of Agencies for Health Technology Assessment and Services of the National Health System (RedETS).

Your experience is essential to improving how patients’ perspectives, needs, and lived experiences are integrated into HTA processes.

**Confidentiality and Data Protection**

In accordance with the General Data Protection Regulation (GDPR), your responses will be treated confidentially and used exclusively for evaluation and continuous quality improvement purposes.

**Section 1. Profile and Participation Context**

**Q1. Full name:**

**Q2. Are you affiliated with a patient organisation or association?**
☐ Yes
☐ No

**Q2a. If yes, please indicate the name of the organisation or association:**

**Q3. Title of the HTA report in which you participated:**

**Q4. How would you describe your role in this HTA report?** (Select one option)

☐ Individual patient
☐ Expert patient
☐ Representative of a patient organisation
☐ Caregiver or family member
☐ Healthcare system user without the assessed condition
☐ Other (please specify): _____________

**Q5. At which stage(s) of the report did you participate?** (Select all that apply)

☐ In defining the protocol or research questions
☐ In a working group meeting
☐ In an individual interview or focus group
☐ In reviewing the draft report
☐ In reviewing a plain language summary
☐ At another stage (please specify): _____________

**Section 2. Recruitment and Initial Information**

**Q6. How were you informed about the opportunity to participate?**
(Select all that apply)

☐ Through a patient association
☐ Through a healthcare professional
☐ Directly by an HTA agency or unit
☐ Through another route (please specify): _____________

**Q7. How did you receive the information or materials related to your participation?**
(Select all that apply)

☐ By email
☐ By telephone
☐ In an in-person meeting
☐ In an online meeting
☐ Other (please specify): _____________

**Q8. Was the information you received before participating clear regarding the purpose of the report and the tasks you were expected to carry out?**
(Select one option only)

☐ Yes
☐ Partially
☐ No

**Q8a. If you answered “Partially” or “No”, please indicate which aspects were difficult to understand:**

**Q9. Were you offered any training or additional information to support your participation?**
☐ Yes
☐ No

**Q9a. If yes, please specify the type of training or information received:**

**Section 3. Experience During Participation**

**Q10. How would you rate the overall communication during your participation?**

☐ Very good
☐ Good
☐ Fair
☐ Poor

**Q11. Please rate the following statements on a scale from 1 to 5, where 1 = Strongly disagree and 5 = Strongly agree:**

• Meeting schedules were compatible with my circumstances
• Face-to-face meeting locations were accessible
• Online meetings were easy to use and accessible
• Email communication was clear and effective
• Telephone communication was clear and effective

**Q12. What difficulties or challenges did you encounter during your participation?**

**Q13. What aspects could be improved to make patient participation easier in the future?**

**Q14. How would you rate your level of satisfaction with the support received from the HTA agency/unit?**
(Select one option only)

☐ Not at all satisfied
☐ Slightly satisfied
☐ Fairly satisfied
☐ Very satisfied

**Q14a. Please explain the reason for your previous answer:**

**Section 4. Perceived Outcomes and Learning**

**Q15. Please rate the following statements on a scale from 1 to 5, where 1 = Strongly disagree and 5 = Strongly agree:**

• I clearly understood what was expected of me
• I was aware of the stages of the report in which I would participate
• My knowledge of HTA increased through this experience
• My participation contributed meaningfully to the report
• Through my participation, I gained a better understanding of the HTA process

**Q16. Were you informed about how your contribution was incorporated into the report?**

☐ Yes
☐ No
☐ I do not know

**Q17. How important do you consider your contribution to have been for the report?**

☐ Not important at all
☐ Slightly important
☐ Quite important
☐ Very important

**Q18. In your view, what did you contribute to the report?**

**Section 5. Post-Participation Experience and Future Interest**

**Q19. Did you receive the final published report resulting from the process in which you participated?**

☐ Yes
☐ No

**Q19a. If yes, did you participate in dissemination activities?**

☐ Yes
☐ No

**Q19b. If yes, please indicate which channels you used:**

**Q20. Would you have liked to receive additional information after your participation?**

☐ Yes
☐ No

**Q20a. If yes, please indicate what information you would have liked to receive:**

**Q21. Would you be interested in participating in future HTA reports or other RedETS activities?**

☐ Yes
☐ No
☐ Not sure

*If you answer “Yes”, your contact details may be included in a regional repository of patients interested in participating in future HTA reports. Inclusion in this repository is entirely voluntary, and you may request removal of your data at any time.*

**End of Questionnaire**

Thank you for your time and valuable contribution. Your responses will help improve the integration of patient perspectives in Health Technology Assessment.

Technical Secretariat
Spanish Network of Agencies for Health Technology Assessment and Services of the National Health System (RedETS)
