# Supplementary material for: Developing and implementing a protocol for evaluating patient involvement in health technology assessment: the experience of the Spanish HTA network (RedETS)
Source: Int J Technol Assess Health Care. 2026 Jul 29;42(1):e76. doi: 10.1017/S0266462326104085 (PMC13735836; doi:10.1017/S0266462326104085)
Supplement: Supplementary file 1 [file S0266462326104085sup001.zip › Supplementary_File_S4_Protocol_Patient_Involvement_RedETS_English.docx]

**S4.- Protocol for the Implementation of the Evaluation of Patient Involvement in RedETS Reports**

**Table of Contents**

1. Introduction
2. General objective
3. Scope
4. Methodology for the evaluation of patient involvement
5. Annual timeline for the evaluation of patient involvement
6. Dissemination and use of results
   Annex 1. Flowchart of the annual process for evaluating patient involvement in HTA reports

**Protocol for the Implementation of the Evaluation of Patient Involvement in RedETS Reports**

**1. Introduction**

The inclusion of the patient perspective in Health Technology Assessment (HTA) reports is an essential aspect to ensure the transparency, legitimacy, and quality of the decisions derived from these reports. Patient involvement provides a complementary perspective to scientific and technical evidence, contributing to ensuring that assessments better reflect the needs, values, and experiences of people affected by a specific health condition.

The evaluation of patient involvement in HTA reports responds to the need to establish systematic mechanisms that allow the analysis, assessment, and continuous improvement of patient engagement in evaluation processes. To date, although there has been a willingness to incorporate the voices of patients into HTA, no specific and structured methodology was available to measure the scope and impact of such participation.

In this context, the Spanish Network of Health Technology Assessment Agencies and NHS Benefits of the National Health System (RedETS) has developed a methodological framework that allows the periodic, systematic, and objective evaluation of patient involvement in HTA processes. This approach seeks to strengthen the quality and relevance of the reports produced by RedETS, ensuring that they adequately reflect the needs and experiences of patients.

**2. General Objective**

To establish a systematic and sustainable framework for the implementation and continuous evaluation of patient involvement in HTA reports produced by the agencies and units that are members of RedETS. This framework will make it possible to assess the scope, quality, and impact of such involvement, identify areas for improvement, and promote a progressive and effective integration of the patient perspective into evaluation processes.

**3. Scope**

This protocol applies to the following actors and activities related to patient involvement in HTA reports:

- **RedETS Secretariat**, responsible for the overall management and coordination of the Network, ensuring the appropriate implementation of the protocol and monitoring its results.
- **Heads of RedETS agencies and units**, responsible for supervising and directing evaluation activities, ensuring the effective integration of patient involvement in HTA reports.
- **Representatives of the Patient and Social Determinants of Health Interest Group (GIP-DSS)**, with a key role in the interpretation of results and the formulation of improvement proposals.
- **Persons coordinating patient involvement in HTA reports**, responsible for leading and organising the inclusion of patients in the evaluation processes carried out in each RedETS agency or unit.
- **Patients and patient associations** who collaborate in HTA reports by contributing their perspectives and experiences; their involvement will be evaluated in order to measure its impact and improve collaboration procedures.

**4. Methodology for the Evaluation of Patient Involvement**

In order to ensure a systematic, homogeneous, and continuous evaluation of patient involvement in HTA reports, a structured process has been designed that combines information collection through questionnaires, analysis of results, and the organisation of periodic meetings for review and decision-making.

**4.1. Composition of the team**

To carry out this process, a working team has been established with clearly defined functions and responsibilities, responsible for coordinating, analysing, and interpreting the evaluation results.

The analysis team is composed of:

- **RedETS Technical Secretariat**, responsible for the overall coordination of the process.
- **One representative of the GIP-DSS from each of the eight agencies or units** that make up RedETS.

**Team responsibilities**

**RedETS Technical Secretariat:**

- Coordinate and supervise the activities of the analysis team.
- Ensure compliance with the objectives established for the evaluation.
- Actively participate in the discussion of results and in the preparation of improvement proposals based on the findings obtained.

**GIP-DSS representatives in each agency/unit:**

- Facilitate the implementation of the evaluation within their respective agency, ensuring the appropriate distribution of questionnaires.
- Contribute to the analysis of data related to the reports produced by their agency, providing contextual and specific knowledge.
- Participate in the interpretation of results and in the definition of improvement proposals aligned with the findings.

**Technical coordination (Eva Reviriego, OSTEBA):**

- Coordinate the process of sending and managing questionnaires to the different target groups.
- Supervise the review, validation, and analysis of the data collected.
- Draft the results reports and prepare improvement proposals derived from the analysis.
- Share consolidated results with the analysis team for review, discussion, and final validation.

**Table 1. GIP-DSS representatives by agency/unit**

| **Agency** | **Name and surname** |
| --- | --- |
| AETSA | Anabel Granja Domínguez |
| AETS-ISCIII | Begoña Rodríguez Ortiz de Salazar / Matilde Palma Ruíz |
| AQuAS | Laura Llinàs Mallol |
| Avalia-T | Paula Cantero Muñoz |
| IACS | Analía Abt Sacks |
| OSTEBA | Eva Reviriego Rodrigo |
| SESCS | Vanesa Ramos García |
| UETS | Gustavo Mora Navarro |

**4.2. Data collection instruments and procedures**

**Platform used**

The evaluation will be managed using the **Encuesta Fácil®** platform, which will enable the secure and centralised distribution, completion, and collection of questionnaires.

**Available questionnaires**

The questionnaires used in the annual evaluation of patient involvement in HTA reports are available online through the following links:

- Questionnaire addressed to Heads of HTA Agencies/Units → [Online access](https://www.encuestafacil.com/respweb/cuestionarios.aspx?EID=2962375)
- Questionnaire addressed to Coordinators of HTA Reports → [Online access](https://www.encuestafacil.com/respweb/cuestionarios.aspx?EID=2962425)
- Questionnaire addressed to Patients → [Online access](https://www.encuestafacil.com/respweb/cuestionarios.aspx?EID=2962426)

In addition, the three questionnaires and their corresponding cover letters are available in Word format for consultation and use by HTA agencies/units.

**Distribution structure and responsibilities**

**a) Technical coordination (Eva Reviriego, OSTEBA)**
Actions:

- Coordinate the overall process of questionnaire distribution and follow-up.
- Send the questionnaire addressed to heads of HTA agencies/units to the RedETS Secretariat for distribution.
- Send the questionnaire addressed to report coordinators to GIP-DSS representatives for dissemination.
- Send the questionnaire addressed to patients to GIP-DSS representatives, who will distribute it to the report coordinators of their respective agencies/units.

**b) RedETS Secretariat**
Actions:

- Distribute the questionnaire addressed to heads of HTA agencies/units for completion.

**c) Heads of HTA agencies/units**
Actions:

- Complete the questionnaire corresponding to their agency/unit.

**d) GIP-DSS representatives**
Actions:

- Collaborate in the distribution and follow-up of the questionnaire addressed to report coordinators within their agency/unit.
- Verify that patient questionnaires have been sent after report publication, in coordination with the responsible technical staff, and report any incidents to the central analysis team.
- Record the actions carried out in a shared follow-up document, ensuring traceability of the process.

**e) Coordinators or technical staff responsible for HTA reports**
Actions:

- Complete one questionnaire per report, regardless of whether patient involvement occurred.
- In cases with patient involvement, send the questionnaire to participating patients immediately after report publication, ensuring the document is accessible.

This action may be reflected in the RedETS quality questionnaire as part of the report closure process, ensuring consistency with RedETS quality objectives.

**Annual evaluation period**

- In the first evaluation, HTA reports published between **January 2024 and February 2025** were analysed.
- In subsequent evaluations, the analysis period will cover reports published between **March of the previous year and February of the current year** (e.g., in 2026, reports published between March 2025 and February 2026 will be evaluated).

**Clarifying note:**
This evaluation focuses exclusively on HTA reports produced within the framework of the RedETS annual work plan. Other products or types of reports (such as emerging technologies, pseudotherapies, or other technical documents) are not currently included but may be considered in future expansions of the protocol.

**Traceability and information control**

To ensure the consistency and completeness of the evaluation process, a cross-check verification table will be incorporated, including:

1. HTA reports published on the RedETS website during the evaluation period.
2. Questionnaires received from heads of agencies, report coordinators (or responsible technical staff), and patients.

This cross-check will allow full traceability of the process, identification of possible inconsistencies or missing information, and confirmation that all published reports have been correctly included in the evaluation.

The linkage between each questionnaire and its corresponding report will be carried out through a controlled coding process in the Excel database, using the official titles or standardised references of the reports published by RedETS.

**Management and access**

The cross-check verification document will be managed by the annual evaluation coordination team, responsible for its updating and safekeeping.
Access to the document will be limited to members of the analysis team within the working group, ensuring confidentiality and exclusive use for evaluation purposes.

**Response deadline**

- The response deadline will be **15 calendar days** from the date of questionnaire distribution.
- After **7 days**, a single reminder will be sent to individuals who have not yet completed the questionnaire, according to the follow-up register.
- In the event of duplicate responses, the coordination team will retain the most recent version by default, verifying the coherence and completeness of the information prior to final validation.

**4.3. Data Analysis and Preparation of the Annual Report**

**Objective of the analysis**

The objective of data analysis is to evaluate patient involvement in HTA reports and to extract conclusions that allow optimisation of the process, identification of areas for improvement, and strengthening of the integration of the patient perspective in health technology assessment.

**Data access and processing**

- Controlled and secure access to the collected data will be ensured in order to protect participants’ privacy and comply with the General Data Protection Regulation (GDPR).
- Data will be used exclusively for the purposes established in this protocol and protected through technical and organisational measures that ensure confidentiality, integrity, and availability.
- Data processing will be carried out by the coordination team, ensuring that only strictly necessary information is collected and used solely for the defined purposes, in accordance with current data protection regulations.

**Review of results and annual analysis meeting**

Each year, an analysis team meeting will be held with the participation of the RedETS Technical Secretariat and the coordination team, with the following objectives:

- Review the results of the three questionnaires (heads of agencies, report coordinators, and patients).
- Identify trends, good practices, and areas for improvement in patient involvement.
- Propose methodological adjustments and strategies to optimise the process in the following evaluation cycle.
- Assess, where appropriate, modifications to this protocol or to the questionnaires used, based on experience from the annual cycle.

**Preparation of the consolidated annual report**

At the end of each evaluation cycle, a consolidated annual results report will be prepared, including:

- A global analysis of the data obtained from the three questionnaires.
- General conclusions on the status and quality of patient involvement in HTA reports.
- Recommendations and improvement proposals based on the findings of the analysis.

The report will be shared with all involved parties, ensuring clear, structured, and timely communication of results and proposed improvement actions.

**4.4. Five-year Study on Patient Involvement**

**Purpose**

Annual evaluation allows monitoring of the evolution of patient involvement in HTA reports and the application of continuous improvements to the process. Complementarily, every five years a five-year case study will be conducted, based on the accumulated results of previous annual evaluations, with the aim of deepening learning, identifying significant changes, and strengthening the integration of the patient perspective in HTA processes.

**Specific objectives**

- Analyse in depth the challenges, progress, and barriers to patient involvement observed over the previous five years.
- Formulate improvement proposals based on findings and reflections derived from accumulated experience and qualitative analysis.

**Frequency**

- The study will be conducted every five years, using as a reference the results of the annual evaluations corresponding to the previous five-year period.
- The first edition of this analysis is scheduled for **2029**.

**Methodology**

**a) Study design**

- The study will adopt a mixed-methods approach, combining qualitative methods with document review.
- Planned techniques include semi-structured interviews, focus groups, and analysis of previous documents and reports.
- The process will be coordinated by Eva Reviriego (OSTEBA), in collaboration with representatives of the agencies that make up the GIP-DSS.
- Sources such as annual questionnaires, evaluation reports on patient involvement, and relevant international literature on patient involvement in HTA will be integrated.

**b) Data analysis**

- Analysis will be conducted collaboratively by Eva Reviriego and representatives of RedETS agencies participating in the GIP-DSS working group.
- Results will be collectively reviewed to ensure consistency, validity, and practical usefulness.

**Presentation and dissemination of results**

- The results of the five-year study will be presented to:
  - Heads and report coordinators of all RedETS agencies and units.
  - The RedETS Technical Secretariat and the GIP-DSS, for discussion, validation, and joint formulation of new strategies to improve patient involvement.
- Conclusions and recommendations derived from the study will be incorporated into the annual methodological review process of RedETS, ensuring continuous system feedback.

**Link with international initiatives**

The five-year study will be enriched by international evidence and experiences, particularly those promoted by HTAi and other international HTA networks focused on the impacts, methods, and processes of patient involvement. These findings will serve as comparative and guiding references, contributing to the alignment of RedETS practices with the most advanced international standards in patient involvement.

The flowchart of the annual process for evaluating patient involvement in HTA reports is provided in **Annex 1 (Figure 1)**.

**5. Annual timeline for the evaluation of patient involvement**

**5.1. Purpose**

To ensure a continuous, systematic, and useful evaluation of patient involvement in HTA reports, an annual timeline has been defined that combines ongoing and periodic activities.

This approach allows regular monitoring, validation of collected information, identification of barriers and opportunities for improvement, and application of strategic adjustments to both methodology and evaluation instruments.

**5.2. Ongoing activities (throughout the year)**

- Sending the questionnaire to participating patients at the time of publication of each report, ensuring that the document is accessible on the RedETS website.
- Continuous monitoring and recording of questionnaire distribution and receipt, in coordination with the central analysis team.

**5.3. Periodic activities (March each year)**

**a) Questionnaire distribution and management**

- Distribution of the questionnaire to heads of HTA agencies/units.
- Distribution of the questionnaire to coordinators or technical staff responsible for HTA reports.
- Response deadline: 15 calendar days from the date of distribution.
- Reminder: a single reminder after 7 days, sent exclusively to individuals who have not responded.

**b) Follow-up and validation (March)**

- Updating the follow-up register, monitoring which reports have completed the questionnaires and which remain pending.
- Verification of traceability by GIP-DSS representatives, in coordination with the central team.

**c) Collection and review (March–April)**

- Collection and preliminary review of received questionnaires.
- Validation of correspondence between published reports and received questionnaires.
- Monitoring compliance with the distribution of questionnaires corresponding to completed reports.
- Preparation of a preliminary analysis identifying initial barriers, good practices, and potential methodological adjustments.

**d) Analysis and annual meeting (May–June)**

- Holding the annual analysis team meeting, with participation of the RedETS Technical Secretariat and GIP-DSS representatives.
- Joint review of results from the three questionnaires.
- Discussion of findings and preparation of methodological and strategic improvement proposals, including possible adjustments to the protocol and data collection instruments.
- Presentation of the draft consolidated annual report.

**e) Review and validation (July–September)**

- Review and validation of the consolidated report by the working group.
- Incorporation of comments and finalisation of materials after feedback.
- Final editing and preparation for public dissemination.

**f) Dissemination (October–November)**

- Publication and communication of final results to all involved parties: heads of agencies/units, GIP-DSS representatives, and other stakeholders.
- Ensuring structured, clear, and timely communication of results and agreed improvement actions.

**Note:** Timelines may be slightly adjusted depending on report completion schedules or team availability. The cycle maintains an annual character, ensuring continuity of the evaluation process.

**5.4. Five-year evaluation**

Every five years, a five-year case study will be conducted, based on accumulated results from annual evaluations, in order to analyse trends and strengthen patient involvement within RedETS.

- First quarter of the fifth year (2029): development of the study with comprehensive analysis of the previous five-year period.
- Second and third quarters: detailed data collection and analysis of the evolution of patient involvement.
- Final quarter: presentation of results to the GIP-DSS, the Technical Secretariat, and other stakeholders to identify trends and define long-term improvement strategies.

(See Section 4.4 for further information on the methodology of the five-year study.)

**5.5. Review and updating of the protocol**

Based on findings from annual and five-year evaluations, necessary adjustments and updates will be made to both this protocol and the questionnaires, ensuring their relevance and continuous improvement.

Modifications will be formally documented through amendments or annexes, depending on their scope. If changes involve a substantial revision of content or structure, a new version of the protocol will be prepared and approved, duly dated and registered.

**6. Dissemination and Use of Results**

**6.1. Purpose**

Dissemination of findings derived from the evaluation of patient involvement is an essential element to ensure transparency, foster collective learning, and promote continuous improvement of the process within RedETS.

**6.2. Products and publications derived from the first cycle**

Following completion of the first annual evaluation cycle (2024–2025), several technical and scientific products have been generated that consolidate the methodological process and its results:

- Protocol for the Implementation of the Evaluation of Patient Involvement in RedETS Reports, revised and validated after the first application cycle.
- Consolidated results report (2024–2025), integrating analyses from the three questionnaires (heads of agencies, report coordinators, and patients) and presenting cross-cutting conclusions and recommendations.
- Updated versions of data collection instruments, adapted based on lessons learned from the first evaluation.
- Scientific article entitled *“Developing and Implementing a Method for Evaluating Patient Involvement in Health Technology Assessment: The Spanish HTA Network (RedETS) Experience”*, submitted for publication to the *International Journal of Technology Assessment in Health Care (IJTAHC)*.

These products will serve as the basis for dissemination of the RedETS methodological model for evaluating patient involvement, as well as for its continuous improvement and international projection.

**6.3. Dissemination channels**

Results and derived products will be shared with stakeholders through the following channels:

- **RedETS**: internal dissemination for discussion of results, planning of future strategies, and exchange of good practices among agencies.
- **Scientific conferences and events**: presentation at national and international forums specialised in HTA and patient involvement.
- **Scientific journals**: publication of articles in journals specialised in methodology, patient involvement, and HTA.
- **Patient organisations**: dissemination of accessible summaries of key findings, addressed to umbrella organisations and patient associations across autonomous communities.

**6.4. Expected impact**

This dissemination process will enable:

- Sharing of knowledge and good practices among agencies and stakeholder groups.
- Validation and strengthening of the methodology used to evaluate patient involvement.
- Promotion of dialogue and collaboration among involved parties, fostering more effective, sustainable, and goal-aligned patient involvement strategies within RedETS.

**Annex 1. Flowchart of the annual process for evaluating patient involvement in HTA reports**
