# Supplementary material for: Developing and implementing a protocol for evaluating patient involvement in health technology assessment: the experience of the Spanish HTA network (RedETS)
Source: Int J Technol Assess Health Care. 2026 Jul 29;42(1):e76. doi: 10.1017/S0266462326104085 (PMC13735836; doi:10.1017/S0266462326104085)
Supplement: Supplementary file 1 [file S0266462326104085sup001.zip › Supplementary_File_S1_Questionnaire_Heads_HTA_Agencies_Revised_English.docx]

**Supplementary File S1. Questionnaire for Heads of HTA Agencies/Units**

Spanish Network of Agencies for Assessing National Health System Technologies and Performance (RedETS)
Annual Evaluation of Patient Involvement in HTA Reports
2025 Version

**Cover Letter**

**Subject:** Annual Questionnaire on Patient Involvement in HTA Reports (RedETS)

Dear Heads of HTA Agencies/Units,

We appreciate your collaboration in completing this annual questionnaire addressed to the Heads of Agencies/Units of the Spanish Network of Agencies for Assessing National Health System Technologies and Performance (RedETS), within the framework of the annual evaluation of patient involvement in HTA reports.

The questionnaire aims to collect updated information on the incorporation of the patient perspective in HTA reports, as well as on the resources, training activities, and strategies implemented within each agency/unit to support patient involvement.

The questionnaire should be completed by the Head of each agency/unit or by a designated representative.

Estimated completion time: approximately 10 minutes.
Responses will be treated confidentially and used exclusively for evaluation and continuous quality improvement purposes.

Deadline for submission: [day] [month].
Access the questionnaire here: [[link](https://www.encuestafacil.com/respweb/cuestionarios.aspx?EID=2962375)]

For further information about the procedure, please consult the Protocol for the Evaluation of Patient Involvement in RedETS (attached).

Thank you in advance for your time and collaboration.

Sincerely,
RedETS Technical Secretariat

**Questionnaire**

This questionnaire is addressed to the Heads of HTA agencies or units and aims to collect updated organisational-level information on the resources, training, and strategies used to support patient involvement in HTA reports.

**Q1. Full name:**

**Q2. Agency/Unit:**

**Q3. Total number of staff working in your agency/unit, by professional profile:**

• Head/Director of the HTA area
• Technical staff involved in HTA report development
• Technical staff exclusively dedicated to other projects
• Documentation support staff
• Administrative staff
• Management/coordination support staff

**Q4. Number of staff involved in patient involvement activities during the past year:**

Indicate the number according to level of involvement:
• Exclusive dedication
• Partial dedication
• Occasional involvement

**Q5. Number of staff who received training or gained experience in patient involvement during the past year:**

☐ None
☐ 1
☐ 2
☐ 3
☐ 4 or more

**Q5a. Type of training or experience received:**

**Q6. Number of staff who received training or gained experience in qualitative research during the past year:**

☐ None
☐ 1
☐ 2
☐ 3
☐ 4 or more

**Q6a. Type of qualitative research training or experience received:**

**Q7. Total number of HTA reports published during the evaluation period:**

**Q8. Number of reports that included effective patient involvement:**

**Q9. Were there attempts to include patient involvement that did not materialise?**

☐ Yes
☐ No

**Q9a. If yes, please indicate the number of reports and the main reasons:**

**Q10. Perceived impact of patient involvement on HTA reports:**

☐ Significant improvement
☐ Moderate improvement
☐ No perceptible change
☐ Don’t know

**Q11. Did your agency/unit organise HTA-related training activities for patients during the past year?**

☐ Yes
☐ No

**Q11a. Number of patients trained:**
☐ None
☐ 1–3
☐ 4–6
☐ 7–9
☐ 10 or more

**Q11b. Number of trained patients who subsequently participated in HTA reports:**
☐ None
☐ 1–3
☐ 4–6
☐ 7–9
☐ 10 or more

**Q12. Were there any costs associated with patient involvement?**

☐ Yes
☐ No
☐ Not applicable

**Q12a. Types of costs covered (select all that apply):**

☐ Travel
☐ Accommodation
☐ Per diems
☐ Financial compensation
☐ Materials
☐ Other (please specify): _____________

**Q13. Did your agency/unit collaborate with other RedETS agencies on qualitative research or patient involvement activities?**

☐ Yes
☐ No

**Q13a. If yes, please specify the agencies involved and the nature of the collaboration:**

**Q14. In your view, what is needed to further strengthen patient involvement in HTA reports?**

**End of Questionnaire**

Thank you for your time and valuable collaboration. Your responses will contribute to improving the integration of patient perspectives in Health Technology Assessment.

Technical Secretariat
Spanish Network of Agencies for Assessing National Health System Technologies and Performance (RedETS)
